# The role of necrosis, acute hypoxia and chronic hypoxia in ^18^F-FMISO PET image contrast: a computational modelling study

**DOI:** 10.1088/1361-6560/61/24/8596

**Published:** 2016-11-23

**Authors:** Daniel R Warren, Mike Partridge

**Affiliations:** CRUK/MRC Oxford Institute for Radiation Oncology, University of Oxford, Old Road Campus Research Building, Roosevelt Drive, Oxford OX3 7DQ, UK; daniel.warren@oncology.ox.ac.uk

**Keywords:** PET, FMISO, hypoxia, computational model

## Abstract

Positron emission tomography (PET) using ^18^F-fluoromisonidazole (FMISO) is a promising technique for imaging tumour hypoxia, and a potential target for radiotherapy dose-painting. However, the relationship between FMISO uptake and oxygen partial pressure (${{P}_{{{\text{O}}_{2}}}}$) is yet to be quantified fully. Tissue oxygenation varies over distances much smaller than clinical PET resolution (<100 *μ*m versus  ∼4 mm), and cyclic variations in tumour perfusion have been observed on timescales shorter than typical FMISO PET studies (∼20 min versus a few hours). Furthermore, tracer uptake may be decreased in voxels containing some degree of necrosis.

This work develops a computational model of FMISO uptake in millimetre-scale tumour regions. Coupled partial differential equations govern the evolution of oxygen and FMISO distributions, and a dynamic vascular source map represents temporal variations in perfusion. Local FMISO binding capacity is modulated by the necrotic fraction. Outputs include spatiotemporal maps of ${{P}_{{{\text{O}}_{2}}}}$ and tracer accumulation, enabling calculation of tissue-to-blood ratios (TBRs) and time-activity curves (TACs) as a function of mean tissue oxygenation.

The model is characterised using experimental data, finding half-maximal FMISO binding at local ${{P}_{{{\text{O}}_{2}}}}$ of 1.4 mmHg (95% CI: 0.3–2.6 mmHg) and half-maximal necrosis at 1.2 mmHg (0.1–4.9 mmHg). Simulations predict a non-linear non-monotonic relationship between FMISO activity (4 hr post-injection) and mean tissue ${{P}_{{{\text{O}}_{2}}}}$ : tracer uptake rises sharply from negligible levels in avascular tissue, peaking at  ∼5 mmHg and declining towards blood activity in well-oxygenated conditions. Greater temporal variation in perfusion increases peak TBRs (range 2.20–5.27) as a result of smaller predicted necrotic fraction, rather than fundamental differences in FMISO accumulation under acute hypoxia. Identical late FMISO uptake can occur in regions with differing ${{P}_{{{\text{O}}_{2}}}}$ and necrotic fraction, but simulated TACs indicate that additional early-phase information may allow discrimination of hypoxic and necrotic signals.

We conclude that a robust approach to FMISO interpretation (and dose-painting prescription) is likely to be based on dynamic PET analysis.

## 1. Introduction

Hypoxia has been implicated as a major cause of local treatment failure in cancers treated with conventional fractionated radiotherapy (Tatum 2006, Vaupel and Mayer 2007). A considerable proportion of hypoxic radioresistance may be attributable to the radiobiological oxygen effect. This phenomenon manifests itself as a 2.5–3.5 times increase in the radiation dose required to achieve a given level of cell kill in hypoxic cells, compared to well-oxygenated cells, and is generally accepted to arise from oxygen acting as a fixing agent for radiation damage (Hall and Giaccia 2006). It has been suggested that patient outcomes may be improved by delivering larger radiation doses to hypoxic regions identified using molecular imaging (Chao *et al* 2001, Ling *et al* 2000, Alber *et al* 2003)—a concept known as hypoxic ‘dose painting’. Positron emission tomography (PET) with the tracer ^18^F-fluoromisonidazole (hereafter denoted FMISO) has shown considerable promise in imaging hypoxia: a number of clinical trials are currently open to investigate the benefits of dose painting using FMISO-derived targets^1^1E.g. ClinicalTrials.gov IDs NCT01576796, NCT02089204, NCT02352792; EudraCT numbers 2010-021139-15 and 2010-021382-78., and the imaging technique has also been investigated to predict patient prognosis or response to therapy (Eschmann *et al* 2005, Rischin 2006, Thorwarth *et al* 2006, Kikuchi *et al* 2011, Zips *et al* 2012). However, there is not yet consensus on the precise quantitative relationship between FMISO image contrast and tumour oxygenation, and most dose painting proposals have taken a pragmatic approach to determine prescriptions (Geets *et al* 2013). Computational modelling may provide additional insights into the quantitative interpretation of FMISO imaging, and will be examined in this work.

### 1.1. Computational modelling of oxygen and FMISO distributions in tumours

There is a large body of literature on the calculation of oxygen distributions arising from multiple microscopic vessels in tumour tissue, most recently reviewed by Toma-Dasu and Dasu (2013). In brief, numerical methods are typically used to solve a partial differential equation for oxygen diffusion with additional consumption term, taking the form:

\begin{eqnarray*}\frac{\partial P}{\partial t}=D{{\nabla}^{2}}P-q(P),\end{eqnarray*}

where *P* is oxygen partial pressure (${{P}_{{{\text{O}}_{2}}}}$), *D* is a diffusion coefficient and *q*(*P*) is a bulk tissue oxygen consumption rate which may vary with ${{P}_{{{\text{O}}_{2}}}}$. Input of oxygen from blood vessels can be mathematically represented either by boundary conditions, or by an additional source term in the equation (Skeldon *et al* 2012). Resulting maps of the oxygen partial pressure have been analyzed to predict tissue radiation sensitivity using models of the oxygen enhancement ratio (OER), providing insights into the biologically-optimal radiotherapy dose (Dasu *et al* 2005, Dasu and Toma-Dasu 2006, Toma-Dasu *et al* 2009b, Powathil *et al* 2012).

Many studies have aimed to obtain biologically relevant results by solving diffusion equations in two dimensions, but a limited number of works have considered the three-dimensional problem. Kelly and Brady (2006) accounted for the third dimension by using a vessel kernel which is averaged over all possible orientations, thereby assuming quasi-linear behaviour. Other authors have taken fully three-dimensional approaches to the oxygenation problem (Beard and Bassingthwaighte 2001, Secomb *et al* 2004, Grimes *et al* 2016b). Two- and three-dimensional approaches have been compared by Espinoza *et al* (2013), with a simple 3D vascular system in which vessels are oriented randomly along one of the Cartesian axes. There was no statistically significant difference in the hypoxic fraction predicted in twenty 2D and 3D simulations with a specified vascular coverage. We have verified that this result applies when vessels are oriented at arbitrary angles, and in simulations of FMISO transport, as shown in appendix A.

Simulated oxygen distributions have been used as a basis for modelling uptake of hypoxia-specific PET contrast agents, including FMISO (Kelly and Brady 2007, Toma-Dasu *et al* 2009a, Dalah *et al* 2010, Mönnich *et al* 2011, Mönnich *et al* 2012, Mönnich *et al* 2013, Wack *et al* 2015). Numerical methods are used to pre-calculate a ${{P}_{{{\text{O}}_{2}}}}$ distribution for tissue with given vasculature, from which a kinetic parameter map for FMISO binding is derived. The parameter map and the original vessel map are used as inputs to a coupled system of reaction–diffusion equations, which describe tracer transport and binding. Approximate solutions are found for the tracer distribution as a function of time, in a similar manner to the oxygen distribution.

Most pre-existing simulations of tracer accumulation (with the notable exception of Mönnich *et al* (2012)) use vessel maps which do not vary over the course of a PET study. However, complex variations in blood vessel perfusion have been observed experimentally on shorter timescales (Chaplin *et al* 1986). Since the radiosensitivity of cells is modulated by the oxygen effect, which depends on the presence of oxygen within a window shorter than 10 ms (Prise *et al* 1999, Hall and Giaccia 2006), a computational model of tumours which reflects these vascular dynamics may be relevant. In addition, necrotic regions of sub-millimetre size have been observed in histological samples of tumours (e.g. Beasley *et al* (2001), Rubin and Casarett (1966), Thomlinson and Gray (1955) and Wijffels *et al* (2000)). Previous works have accounted for this using an FMISO binding relation that is a function of instantaneous local ${{P}_{{{\text{O}}_{2}}}}$, with the effective binding rate increasing as ${{P}_{{{\text{O}}_{2}}}}$ decreases, reaching a peak at low oxygen levels, before dropping to zero in anoxia due to hypoxic cell death. However, necrosis develops over periods of time that are longer than the fluctuations in perfusion (Franko and Sutherland 1978), so a model that decouples the two processes may provide additional insight in the interpretation of clinical FMISO images. Dynamic vasculature and separate consideration of hypoxic necrosis are key features of the model developed herein.

### 1.2. Vascular dynamics and acute hypoxia

There is considerable evidence that vascular perfusion in some tumours varies over time. Studies in which two independent vascular labels are injected into animals, separated by a period of minutes, do not show complete co-localisation of markers after tumour excision (Chaplin *et al* 1987, Trotter *et al* 1989). Mismatches are typically observed in 10–20% of vessels mapped 20 min apart, although in some cases mismatches in excess of 50% are observed (Durand and Lepard 1995). Similarly, histological comparison of perfusion markers and vascular structure stains in preclinical models have shown instantaneous perfused fractions in the range 20–85% (typically  >55%) (Bernsen *et al* 1995).

Local perfusion has been examined in superficial tumours by using laser Doppler flowmetry in a clinical setting (Pigott *et al* 1996), with perfusion changes greater than  ±50% being observed in 54% of tumour microregions (approx. volume 0.01 mm^3^) over a 1 h period. Fluorescence videomicroscopy has been used to monitor window-chamber tumours in rats, showing a wide variation in individual vessels’ red blood cell flux over 1 h (apex/nadir ratios 1.5–10) (Kimura *et al* 1996). Under similar conditions, the ${{P}_{{{\text{O}}_{2}}}}$ in proximity to a vessel has been found to correlate well with the red blood cell flux therein (Lanzen 2006). Temporal variations in local oxygenation have also been observed in pre-clinical (e.g. Cardenas-Navia *et al* (2008)) and human tumours (e.g. Whittle *et al* (2010)) using continuous electrode measurements—it is believed that local changes in perfusion may be a major contributing factor to these, and therefore to transient (or acute/cyclic) hypoxia (Dewhirst *et al* 2008).

A handful of pre-existing studies have examined oxygen transport calculations in the case of dynamic vasculature, assessing the magnitude of resulting variations in ${{P}_{{{\text{O}}_{2}}}}$. Secomb *et al* (1995) used a Green’s function method to calculate oxygen distributions in tissue with a measured vascular network. A global increase in blood flow of approximately 70% was sufficient to reduce the predicted hypoxic fraction (defined as ${{P}_{{{\text{O}}_{2}}}}&lt;3$ mmHg) from 34% to zero. This approach was extended in Kimura *et al* (1996), by using temporally-resolved measurements of red blood cell flux measurements to define individual vessel flow rates and performing calculations for different phases of acute hypoxia. For a 0.02 mm^3^ tumour region containing 22 vessel segments, the method estimated 25% of the volume was chronically hypoxic and 35% was transiently hypoxic. Dasu *et al* (2003) simulated oxygen distributions using a finite element method and vessel maps derived from intervascular distance measurements in tumours and normal tissues. Acute hypoxia was modelled by randomly closing 25% of vessels, causing hypoxic fractions (${{P}_{{{\text{O}}_{2}}}}&lt;2.5$ mmHg) to vary in the range 6.4–27.3% depending on the position of the closed vessels, compared to 0.2% with all vessels open. This model has subsequently been applied to calculate the radiobiological effects of acute hypoxia (Dasu *et al* 2005).

To the authors’ knowledge, the only prior simulations of FMISO binding in a dynamic vascular scenario have been presented by Mönnich *et al* (2012). A source distribution was derived from a histological section, and two dynamic situations were examined: sinusoidal fluctuation of ${{P}_{{{\text{O}}_{2}}}}$ in the range 30–40 mmHg in all vessels, and complete collapse of blood flow in a region comprising approximately 5% of the domain. Compared to the static scenario (intravascular ${{P}_{{{\text{O}}_{2}}}}=40$ mmHg), a decrease in spatial and temporal mean tissue ${{P}_{{{\text{O}}_{2}}}}$ was observed, with the greatest local variations occurring at both extremes of the histogram (i.e. in the most hypoxic and least hypoxic bins). FMISO accumulated at a somewhat greater rate in the dynamic scenario than the static one: the sinusoidal variations led to approximately 13% increase in activity at 4 h, and an additional 7% resulted from supply collapse. Mönnich’s work provides insight into changes in FMISO contrast resulting from temporal ${{P}_{{{\text{O}}_{2}}}}$ variations that are highly-correlated over an area  ∼1 mm^2^. In this work, we will seek to perform a complementary study, by examining the effects of random fluctuations in perfusion on a sub-millimetre scale.

### 1.3. FMISO binding models

FMISO is an analogue of misonidazole, in which a methoxy group on the alkyl side chain has been replaced by a fluorine atom, specifically ^18^F for PET imaging (Rasey *et al* 1987). Misonidazole has been shown to bind specifically to cells that are hypoxic yet viable, demonstrated by the ‘halo’ of binding seen surrounding the anoxic core of large multicellular tumour spheroids (Franko and Chapman 1982, Rasey *et al* 1985). FMISO binding has been described as a result of two chemical reductions in the intracellular space, which produce a highly reactive product that quickly binds to nearby organic macromolecules (Padhani *et al* 2007). The first reduction step requires an active electron transport chain, so it does not occur in dead cells; the second step is competitive with oxygen and leads to the hypoxia-specific binding characteristic.

A mathematical model of FMISO binding kinetics in the presence of varying oxygen concentration has been proposed by Casciari *et al* (1995). This multi-compartment model allows for reaction products that are bound irreversibly, or temporarily retained, and makes predictions which fit time activity curves (TACs) for both human and rat tumours. It has been calibrated using measurements in V79 cell monolayers (Casciari and Rasey 1995), and has been adopted, with the additional assumption of irreversible binding, in the previously-cited works by Kelly and Mönnich. A parameter-heavy electrochemical model also exists, as described by Bowen *et al* (2011).

Whilst the Casciari model reflects the complex reaction scheme of FMISO in cells, there are challenges in adopting it to predict spatiotemporal FMISO distributions in clinical tumours. In particular, it is not possible to use measured blood time activity curves as a proxy for the tracer present in vessels, since the model assumes blood also contains radioactive diffusible reduction products. However, Casciari’s analysis of patient blood samples 120–160 min post-injection (p.i.) suggests only 15% of blood activity arises from reduction products. We therefore choose to calibrate a simpler irreversible binding model to experimental data, with the expectation that the behaviour of this non-dominant compartment will be partly reflected in the fitted parameter values, and it will only have a minor influence on model behaviour.

### 1.4. Statement of purpose

This work presents a computational model, calibrated using experimental data, to simulate oxygen and misonidazole transport within tumour tissue. The size of the simulation domain is comparable to the resolution of a typical PET scanner (∼4 mm), which cannot directly capture all radiobiologically-important heterogeneity in oxygenation (Busk *et al* 2008, 2009); simulation elements are smaller than characteristic distances for oxygen supply in tissue (∼100–200 *μ*m).

Previous simulations of oxygen transport and FMISO binding have either been performed in static vasculature, or in vessel maps with temporally-coordinated variations in ${{P}_{{{\text{O}}_{2}}}}$. The approach here differs by considering tissue with vessels whose individual perfusion status varies randomly in an uncoordinated fashion. Additionally, the model is formulated such that FMISO binding in tissue is dependent on an underlying map of living cells, which reflects the onset of necrosis after long periods of time at very low oxygen partial pressure (${{P}_{{{\text{O}}_{2}}}}$).

The objective is to identify model behaviour which may be relevant when developing a strategy for dose painting or hypoxia assessment using FMISO. In particular, the primary aim is to identify the extent to which perfusion fluctuations affect observed FMISO image contrast, and its interpretation in terms of tissue oxygenation.

## 2. Materials and methods

### 2.1. Modelling approach

The model described herein calculates concentrations of oxygen and misonidazole in cancer cells as a function of time and position. It can be applied to cells grown as an avascular spheroid *in vitro*, or to cells that grow *in vivo* as part of a solid tumour. In this work, the model will be characterised using data measured in the former situation, and applied to make predictions for the latter situation.

**Figure 1.**
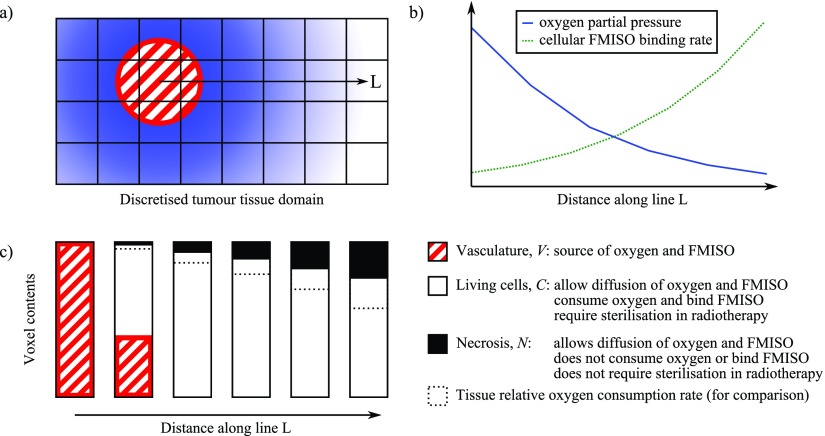
Schematic of the proposed model for oxygen and misonidazole concentrations in tumour. (a) Representation of a single blood vessel position within the discretised tissue domain. The line L designates a path extending in a radial direction, passing through six volume elements. Blue shading represents the oxygen concentration in tissue. (b) Illustrative profiles of oxygen partial pressure and FMISO binding rate in living tissue as a function of distance along L. (c) Volume fractions of the three modelled tissue components (vasculature, living cells and necrosis) in the six voxels intersected by L. The dashed bar, as measured from the bottom, represents the relative oxygen consumption rate as predicted by the Michaelis–Menten relationship employed in the model.

A schematic of the main features of the model is presented in figure 1. The first step of the process is generation of a simulation domain, comprising many discrete volume elements. In the case of avascular spheroids a spherical domain is sub-divided into shells of equal thickness. Solid tumours are represented by a rectangular cuboidal domain sub-divided into cubes.

Each volume element is allowed to contain varying proportions of living cells (*C*), vasculature (*V*) and necrosis (*N*  =  1  −  *C*  −  *V*). Vasculature supplies tissue with oxygen, which is consumed by living cells, and misonidazole, which may be bound in living cells. Regions of necrosis do not consume or bind either molecule but still allow for them to diffuse freely. *C* and *N* may vary with time, as a result of a dynamic (i.e. time-varying) vasculature or because of changes induced by radiotherapy.

A series of coupled partial differential equations govern the evolution of oxygen and misonidazole distributions in the domain over time, in a similar manner to many works cited in section 1.1. Numerical methods are used to find approximate solutions to these equations for a pre-defined dynamic vasculature and misonidazole input function.

Each of the various elements of the model is described in more detail below.

#### 2.1.1. Vasculature.

Spheroids have no vasculature, so vascular fraction *V*  =  0 at all positions. In this case oxygen and misonidazole sources are represented by fixed boundary conditions setting ${{P}_{{{\text{O}}_{2}}}}$ and misonidazole concentration at the spheroid surface.

In solid tumour, a two-dimensional vascular map *V*(*x*,*y*) is generated. Individual vessels *V*_*i*_(*x*,*y*) are modelled as rasterized cylinders oriented perpendicular to the simulation plane, with central positions randomly sampled from a uniform distribution and fixed radius *r*_*v*_ defined by the user. A static vasculature is then specified by

\begin{eqnarray*}{{V}_{\text{S}}}(x,y)=\underset{i}{\sum}\,{{V}_{i}}(x,y),\end{eqnarray*}

or a dynamic vasculature by

\begin{eqnarray*}{{V}_{\text{D}}}(x,y,t)=\underset{i}{\sum}\,{{f}_{i}}(t)\centerdot {{V}_{i}}(x,y),\end{eqnarray*}

where *f*_*i*_(*t*) is a function representing the perfusion status of vessel *i* at time *t* and takes values in the the range [0,1]. In the event of overlapping vessels, the sums are constrained such that *V* never takes a value greater than 1.

Vessel perfusion status *f*_*i*_ is modelled as a Boolean function (0  =  non-perfused, 1  =  perfused). *f*_*i*_ has a cyclic behaviour: the perfusion status of each vessel persists for a time randomly sampled from a normal distribution, after which it switches to the other perfusion status. The switching behaviour is repeated *ad infinitum* with a different randomly-generated duration for each iteration. Simulations are performed with an average fluctuation period of 20 min (i.e. open/closed states persist for 10 min on average) and coefficient of variation ($\frac{\sigma}{\mu}$) of 20%, unless otherwise stated, although variations this parameter are also examined. At the beginning of a simulation, individual *f*_*i*_ values are randomly allocated such that a specified fraction of vessels are perfused, and a random time offset is assigned to each vessel to desynchronize their cycles. Perfused fractions in the range 50–100% are examined, reflecting the approximate range of instantaneous perfused fractions and vessel perfusion mismatches reported in the literature.

The model has been implemented in 3D; however, this paper will consider the results of 2D simulations, since the statistical properties of 2D simulations with a 2D vascular model have been shown equivalent to those of 3D simulations with a 3D vascular model (Espinoza *et al* (2013) and appendix A of this work).

#### 2.1.2. Oxygen diffusion and consumption.

The following equation is solved to find oxygen partial pressure *P* within the tissue as a function of position and time *t*:

1 \begin{eqnarray*}\frac{\partial P}{\partial t}=(C+N)\centerdot D_{t}^{{{O}_{2}}}\centerdot {{\nabla}^{2}}P-C\centerdot q(P)+V(t)\centerdot {{f}_{v,{{O}_{2}}}}(P)\end{eqnarray*}

\begin{eqnarray*}q(P)={{q}_{\text{max}}}\frac{P}{P+{{P}_{50,q}}}\end{eqnarray*}

\begin{eqnarray*}{{f}_{v,{{O}_{2}}}}(P)=\frac{D_{v}^{{{O}_{2}}}}{{{r}_{v}}\centerdot {{d}_{v}}}\centerdot \left({{P}_{v}}-P\right)\end{eqnarray*}

*q*(*P*) is a consumption term following Michaelis–Menten kinetics. The source term ${{f}_{v,{{O}_{2}}}}(P)$ is necessary to properly model intravascular resistance, and is constructed from an approximate solution to the diffusion equation across a cylindrical shell (representing the vessel wall). Constants are defined in table 1. *C*, *N*, *V*(*t*) and *P* (and any functions thereof) vary with position. This set of equations is an adapted form of those used by Kelly and Brady (2006) and discussed by Skeldon *et al* (2012).

**Table 1.** Definition and estimated values of parameters used in equation (1). The calibration process described in section 2.2 determines cell-line-specific values for parameters marked ^*^.

Symbol	Meaning	Estimated value	Reference
$D_{t}^{{{O}_{2}}}$	Diffusion coefficient of oxygen in bulk tissue	2 × 10^−9^ m^2^ s^−1^	Tannock (1972)
*q*_max_	Maximum tissue oxygen consumption rate	15 mmHg s^−1^^*^	Dasu *et al* (2003)
*P*_50,*q*_	Partial pressure of oxygen for 50% drop in tissue consumption rate	2.5 mmHg ^*^	Dasu *et al* (2003)
$D_{v}^{{{O}_{2}}}$	Diffusion coefficient of oxygen in vessel wall	2 × 10^−10^ m^2^ s^−1^	Sasaki *et al* (2012)
*r*_*v*_	Vessel radius	10 *μ*m	Kelly and Brady (2006)
*d*_*v*_	Vessel wall thickness	1 *μ*m	Kelly and Brady (2006)
*P*_*v*_	Partial pressure of oxygen inside vessels	40 mmHg	Dasu *et al* (2003)

Oxygen consumption in vessel walls is neglected, with experimental data (Sasaki *et al* 2012) suggesting approximately 1% reduction in the source term for vessels with the dimensions studied here.

#### 2.1.3. Hypoxic necrosis.

Cells are expected to have reduced viability in resource-starved environments. Low oxygen concentrations have been shown to cause cell death over prolonged periods—reported response times range from a few hours to approximately a week (Franko and Sutherland 1978, Shimizu *et al* 1996)—and necrosis develops in the centre of large tumour spheroids. In this model, the quantity *N* will be used to represent the corresponding reduction in cells available to bind FMISO. This will be termed the necrotic fraction, but it encompasses all mechanisms that have the same effect (not just necrotic cell death).

We make the assumption that oxygen availability is the primary determinant of *N*. It should be noted, however, that changes in glucose concentration have been seen to modulate the necrotic fraction in tumour spheroids (Freyer and Sutherland 1986, Luk and Sutherland 1987). This phenomenon might be explained by glucose-induced changes in oxygen consumption rate, or by a lack of glucose directly limiting cell viability. Even in the latter case, we expect that ${{P}_{{{\text{O}}_{2}}}}$ will be an adequate proxy for the development of necrosis: oxygen and glucose are both distributed by the vasculature and consumed by cells *in vivo*, so their concentration profiles should be highly correlated.

Local oxygen partial pressure varies as a function of time in the dynamic vascular model. We assume that the timescale of changes in vascular perfusion is sufficiently short, such that *N* as a function of position can be characterised using the temporal average of the ${{P}_{{{\text{O}}_{2}}}}$ map, $\langle P\rangle $. We follow Kelly and Brady (2007) in modelling cell death as a saturable process, such that *N* takes the functional form:

2 \begin{eqnarray*}N=1-\frac{\langle P\rangle}{\langle P\rangle +{{P}_{50,n}}},\end{eqnarray*}

where *P*_50,*n*_ is the temporal mean ${{P}_{{{\text{O}}_{2}}}}$ that reduces the density of living cells by 50%. The value of this parameter will be determined from spheroid data.

Death is considered to be the last stage of a cell’s response to hypoxia, being preceded by a reduction in oxygen consumption as a result of protective processes. Therefore, the Michaelis–Menten consumption term *q*(*P*) accounts for decreased oxygen consumption due to cell death in the static vascular model. The same rule is applied to dynamic vasculature, since the model behaviour is approximately linear and therefore the value of $\langle q(P)\rangle $ and $q(\langle P\rangle )$ will be similar over the simulation time-frame. Misonidazole binding will be modulated by 1  −  *N*.

#### 2.1.4. Misonidazole diffusion and binding.

The model allows misonidazole to be present in one of three compartments: vascular (unbound but spatially-constrained, concentration denoted *M*_*v*_), bulk tissue (unbound and freely-diffusing, concentration denoted *M*_*f*_) and irreversibly bound (spatially-constrained, concentration denoted *M*_*b*_). For the latter two compartments, concentrations within tissue as a function of position and time *t* are given by the following pair of equations:

3 \begin{eqnarray*}\frac{\partial {{M}_{f}}}{\partial t}=(C+N)\centerdot D_{t}^{M}\centerdot {{\nabla}^{2}}{{M}_{f}}-C\centerdot {{k}_{b}}(P)\centerdot {{M}_{f}}+V(t)\centerdot {{f}_{s,\,M}}\left({{M}_{f}}\right)\end{eqnarray*}

4 \begin{eqnarray*}\frac{\partial {{M}_{b}}}{\partial t}=C\centerdot {{k}_{b}}(P)\centerdot {{M}_{f}}\end{eqnarray*}

\begin{eqnarray*}{{f}_{s,M}}(P)=\frac{D_{v}^{M}}{{{r}_{v}}\centerdot {{d}_{v}}}\centerdot \left[{{M}_{v}}(t)-{{M}_{f}}\right]\end{eqnarray*}

*k*_*b*_(*P*) is the oxygen-specific misonidazole binding rate, which will be determined from spheroid data. The source term *f*_*s*,*M*_(*P*) is constructed in a similar manner to the oxygen source term and is modulated by the same vascular perfusion function *V*(*t*), reflecting our assumption that the vasculature acts as both a source of oxygen and FMISO. *M*_*v*_(*t*) is the vascular misonidazole input function (a user-defined input). It is also possible for *f*_*s*,*M*_(*P*) to act as a sink term if the free misonidazole concentration is greater than that in the input function. We assume that the misonidazole diffusion coefficient is the same in vessel wall and tissue i.e. $D_{t}^{M}=D_{v}^{M}=5.5\times {{10}^{-11}}$ m^2^ s^−1^.

This set of equations is an adapted form of those used by Kelly and Brady (2006) and discussed by Skeldon *et al* (2012). *C*, *N*, *V*(*t*), *P*, *M*_*f*_ and *M*_*b*_ (and any functions thereof) vary with position.

We take the functional form of *k*_*b*_(*P*) from the rate constant for bound product in the reaction scheme described by Casciari *et al* (1995).

5 \begin{eqnarray*}{{k}_{b}}(P)=\frac{{{k}_{b,0}}\centerdot {{P}_{50,b}}}{P+{{P}_{50,b}}}\end{eqnarray*}

The maximum binding rate *k*_*b*,0_ and ${{P}_{{{\text{O}}_{2}}}}$ for 50% reduction in binding *P*_50,*b*_ will be determined from spheroid data.

### 2.2. Calibration using spheroid data

#### 2.2.1. Input data.

The model is calibrated using misonidazole uptake data available in the scientific literature. In particular, we rely upon the data of Gross *et al* (1995) and Raleigh *et al* (1985). In these studies, ${{P}_{{{\text{O}}_{2}}}}$ profiles were measured in avascular spheroids of EMT6 cells. These spheroids were bathed in tritiated misonidazole, sectioned and subjected to autoradiography, allowing uptake to be quantified by the density of activated grains along radii. However, there is one major methodological difference between the two works: Gross scored grain density in all regions, whilst Raleigh only counted grains in non-necrotic regions. The effect of necrosis may therefore be isolated by comparison of the two authors’ results. Experimental details for both of these studies are summarized in table 2.

**Table 2.** Summary of key experimental conditions for the EMT6 spheroid misonidazole binding studies used to calibrate the model. Quantities ${{\eta}_{a}}$ and ${{\eta}_{l}}$ are calculated as described in appendix B.

	Raleigh *et al* (1985)	Gross *et al* (1995)
Necrotic regions scored?	No	Yes
Mean spheroid radius, *r*_*s*_	660 *μ*m^a^	433.5 *μ*m
Oxygen conditions	0.4% / air	air
Section thickness, *d*_*s*_	4 *μ*m	3.5 *μ*m^b^
Autoradiographic efficiency, ${{\eta}_{a}}$	0.0671	0.0763
Misonidazole concentration, *M*_0_	25 *μ*M	50 *μ*M
Labelling efficiency, ${{\eta}_{l}}$	0.0024	0.0031
Misonidazole incubation time, *t*_bath_	3 h	90 min
Autoradiography exposure time, *t*_exp_	16 d	60 d

a Given in cited work Franko and Koch (1983).

b Given in cited work Bourrat-Floeck *et al* (1991).

#### 2.2.2. Fitting methodology.

Autoradiographic grain densities were converted to intracellular concentrations by the method detailed in appendix B. Energy spectra for electrons emitted in beta decay of ^3^H nuclei were obtained from Mantel (1972), and electron range in water as a function of energy was extracted from Meesungnoen *et al* (2002).

The differential equations (1), (3) and (4) were solved in one dimension (spherical radius) using the MATLAB (Mathworks, Natick, MA, USA) function pdepe. Optimal values for the parameters *q*_max_, *P*_50,*q*_, *k*_*b*,0_, *P*_50,*b*_ and *P*_50,*n*_ were found by minimizing the sum of the square differences between modelled and experimental misonidazole concentration profiles, using the Levenberg-Marquardt algorithm.

The first stage was a two-parameter fit for oxygen consumption parameters *q*_max_ and *P*_50,*q*_ using Gross’ measured ${{P}_{{{\text{O}}_{2}}}}$ profile. A fixed boundary condition was imposed on equation (1), setting the spheroid surface ${{P}_{{{\text{O}}_{2}}}}$ to the measured value of 102 mmHg, which is consistent with reports of EMT6 spheroid surface oxygenation where stirred medium is in contact with air (Mueller-Klieser and Sutherland 1982).

The second stage is a fit for misonidazole binding parameters in non-necrotic tissues, so it is assumed that *N*  =  0. The parameters determined in the first stage fit are used to generate ${{P}_{{{\text{O}}_{2}}}}$ profiles for Raleigh’s spheroids in air and 4000 ppm oxygen, providing inputs to equations (3) and (4). A fixed boundary condition is imposed, setting the spheroid surface misonidazole concentration to the value used in the experiment. A two-parameter fit is then performed to determine *k*_*b*,0_ and *P*_50,*b*_ by comparing simulated *M*_*b*_ to Raleigh’s two measured misonidazole profiles; both profiles are weighted equally, and the function minimized is the sum of the square differences relative to the respective profile’s mean.

The third stage uses Gross’ misonidazole profiles to fit the hypoxic necrosis parameter *P*_50,*n*_. An oxygen profile is generated, and used as an input to the misonidazole equations with *N* given by equation (2) and the previously determined binding parameters. A single- parameter fit to Gross’ measured misonidazole profile then determines *P*_50,*n*_.

A sanity check is also performed by carrying out a five-parameter fit directly to the Gross data, with parameters initialized using the values found in the three-stage fit. For each fit, parameter confidence intervals are estimated by the bootstrap resampling method, using 1000 randomly-drawn samples. For the three-stage fit, each bootstrap iteration simulates every stage of the fitting process using a single set of resampled data.

### 2.3. Predictive simulations for bulk tumour

Simulations in bulk tumour are performed using an in-house MATLAB program, specifically developed to allow simultaneous calculation of oxygen and FMISO distributions as a function of time in temporally-varying vasculature. This software generates a vascularised tissue domain, and uses finite difference methods to find the approximate time evolution of solutions to equations (1), (3) and (4) therein.

Regions within bulk tumour are modelled as two-dimensional domains with dimension 1 mm  ×  1 mm, comprising volume elements of size 10 *μ*m  ×  10 *μ*m. Other authors have used computational domains of similar dimensions (Dasu *et al* 2003, Kelly and Brady 2006, Skeldon *et al* 2012, Espinoza *et al* 2013), which are justifiable since they are sufficiently large compared to the tissue region influenced by an individual blood vessel (∼100–150 *μ*m).

Vessels are represented by rasterized cylinders of radius 10 *μ*m running perpendicular to the tissue plane, passing through points selected randomly from a uniform distribution. Multiple vascular maps are generated with different mean vessel densities in the range 1–1000 vessels mm^−2^, covering the typical (approx. 10–300 vessels mm^−2^ (Weidner 1995)) and extreme (MacLennan and Bostwick 1995, Gulubova and Vlaykova 2009) values reported in the literature. Each vessel’s geometry is stored independently for the dynamic vascular model, allowing vessel maps to vary as a function of time.

Model parameters adopted are given in table 1, with additional parameters determined by the calibration process described in the previous section. All perfused vessels in the simulation domain are assumed to contain the same concentration of tracer at a particular moment in time, given by the FMISO input function *M*_*v*_(*t*). This input function represents the injection and longitudinal dispersion of the radioactive bolus, and tracer metabolism, all of which are considered to occur outside the simulation domain. *M*_*v*_(*t*) is specified in terms of blood activity, attenuation-corrected to reflect activity at the time of injection. The function used in this work was extracted from an arterial region in a 4 h dynamic FMISO PET study, and was interpolated between frames by fitting a bi-exponential decay function (Thorwarth *et al* 2005).

In the case of the static vascular function *V*_S_, simulations are performed consecutively; a steady-state oxygen distribution is found, which is then used to calculate *N* and *k*_*b*_ maps for a subsequent FMISO simulation in the same vasculature. In the case of a dynamic vascular function *V*_D_, the oxygen and FMISO simulations are coupled. The temporal average ${{P}_{{{\text{O}}_{2}}}}$ map $\langle P\rangle $ is estimated by performing an initial 1 h simulation without FMISO, and used to calculate *N*, which is considered static over the simulation time-frame^2^2The mean convergence error in *N*, arising as a result of the relatively short simulation time, was assessed by comparison to 48 h simulations and found to be  <2%.. This necrosis map is used as an input to the full 4 h simulation with FMISO, in which *k*_*b*_ is recalculated at each time-point based on the instantaneous ${{P}_{{{\text{O}}_{2}}}}$.

The finite difference calculations are accelerated by use of an adaptive time step, whose length is adjusted such that the ${{P}_{{{\text{O}}_{2}}}}$ and FMISO concentration in any voxel change by no more than 0.1 mmHg or 0.1 kBq ml^−1^ (decay-corrected) respectively.

## 3. Results

### 3.1. Model parameterisation

**Figure 2.**
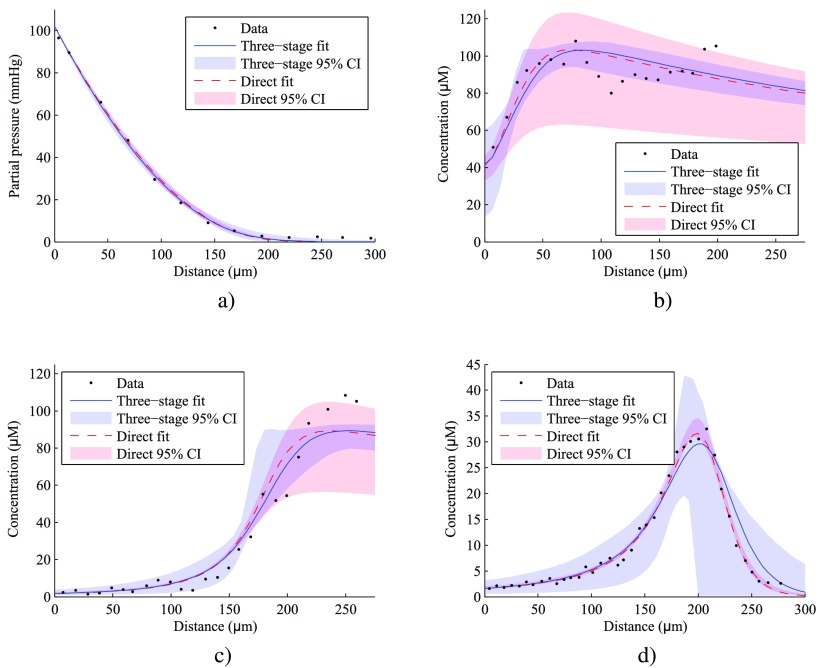
Predictions of the misonidazole binding model for the tumour spheroids described in Raleigh 1985 and Gross 1995, plotted alongside original data. Fitted parameters are as given in table 3. In the three-stage fit, individual datasets were used to determine the parameters for oxygen consumption (a), misonidazole binding ((b) and (c)), and hypoxic necrosis (d). In the direct fit, all parameters were determined from a single dataset (d). Regions representing 95% confidence intervals are derived from bootstrap resampling as in table 3. (a) Oxygen profile, Gross 1995. (b) Bound misonidazole excluding necrosis, Raleigh 1985 (3000 ppm oxygen). (c) Bound misonidazole excluding necrosis, Raleigh 1985 (air). (d) Bound misonidazole including necrosis, Gross 1995.

Table 3 gives optimal values and bootstrapped confidence intervals for the parameters of the oxygen consumption and misonidazole binding model, as fitted to the data of Raleigh and Gross. The direct and three-stage fitting methods produce parameter estimates which are consistent with each other. Oxygen and misonidazole profiles predicted by the model are plotted in figure 2, alongside the source data. Best-fit profiles follow the shape of the original data, predicting the distance for half-maximal binding within 10 *μ*m and showing a maximum deviation of approximately 20% peak misonidazole binding.

**Table 3.** Fitted parameters for the oxygen diffusion and misonidazole binding model. Values are given for a three-stage fit using data from both Raleigh 1985 and Gross 1995, and for a direct five-parameter fit to data from Gross 1995 only. The fitting methodology is described in section 2.2.2. Confidence intervals are estimated using the bootstrap resampling method with 1000 random samples—in the case of the three-stage fit, each bootstrap simulates all stages of the fitting process for a random sample of the input data, leading to broader confidence intervals.

	Three-stage fit	95% CI	Direct fit	95% CI
Maximum oxygen consumption rate, *q*_max_ ($\text{mmHg}\centerdot {{\text{s}}^{-1}}$)	17.5	15.3–25.1	16.3	15.3–17.9
${{P}_{{{\text{O}}_{2}}}}$ for 50% drop in consumption, *P*_50,*q*_ (mmHg)	2.7	0.0–12.5	1.6	1.2–2.1
Maximum misonidazole binding rate, *k*_*b*,0_ ($\times {{10}^{-4}}{{\text{s}}^{-1}}$)	4.5	3.9–4.9	4.4	2.5–5.3
${{P}_{{{\text{O}}_{2}}}}$ for 50% drop in binding, *P*_50,*b*_ (mmHg)	1.4	0.3–2.6	1.4	1.1–2.5
${{P}_{{{\text{O}}_{2}}}}$ for 50% necrosis, *P*_50,*n*_ (mmHg)	1.2	0.1–4.9	1.0	0.4–1.2

The best agreement is observed between the direct five-parameter fit and the Gross data (figure 2(d)), which is also associated with the narrowest confidence intervals. This constitutes a best-case scenario: model fitting and validation are being performed upon the same dataset, and the fitting procedure has the greatest degrees of freedom. The three-stage fit gives broader confidence intervals in this dataset and slightly overestimates the depth at which bound misonidazole concentration peaks. This reduction in fit quality is a result of additional constraints being imposed on the model by the other datasets—four of the five parameters have been determined before the three-stage fit considers the Gross misonidazole profile.

The Raleigh dataset is described comparably well by best-fit lines of both fitting approaches. A greater degree of scatter is evident compared to the Gross data, especially in figure 2(b) – a possible consequence of the small volume of non-necrotic material available for analysis at greater depths, or uncertainties in the classification of necrosis. It is notable that both lines underestimate the maximum plateau height in figure 2(c) by  ∼10%, which could indicate a systematic error in an unfitted input parameter (e.g. in table 2) or suggest a departure in the functional form of *k*_*b*,0_ from equation (5) at very low ${{P}_{{{\text{O}}_{2}}}}$. In the most hypoxic regions of Raleigh’s spheroids, confidence intervals are considerably narrower for the three-stage fit compared to the direct fit, which has less predictive power since it was calibrated using data with necrosis.

In general, the three-stage fit would be expected to lead to more reliable predictions than the direct fit, given that two independent datasets are used in calibration. This is supported by the observation that the confidence interval widths are more consistent across datasets. We therefore adopted best-fit parameters from the three-stage fit for the subsequent bulk tumour simulations.

The maximum binding rate in the model for spheroids with necrosis occurs at:

\begin{eqnarray*}\frac{\text{d}}{\text{d}P}\left({{k}_{0,b}}\centerdot \frac{{{P}_{50,b}}}{P+{{P}_{50,b}}}\centerdot \frac{P}{P+{{P}_{50,n}}}\right){{|}_{P={{P}_{{{k}_{\text{max}}}}}}}\,\,=0\end{eqnarray*}

\begin{eqnarray*}{{P}_{{{k}_{\text{max}}}}}=\sqrt{{{P}_{50,b}}\centerdot {{P}_{50,n}}},\quad {{k}_{\text{max}}}={{k}_{0,b}}\centerdot \frac{{{P}_{50,b}}}{{{\left(\sqrt{{{P}_{50,b}}}+\sqrt{{{P}_{50,n}}}\right)}^{2}}}.\end{eqnarray*}

With the calibrated parameter values, ${{k}_{\text{max}}}=1.2\times {{10}^{-4}}{{\text{s}}^{-1}}$.

### Predicted ^18^F-MISO uptake versus mean ${{P}_{{{\text{O}}_{2}}}}$ in bulk tumour

3.2.

#### 3.2.1. Static uptake maps.

**Figure 3.**
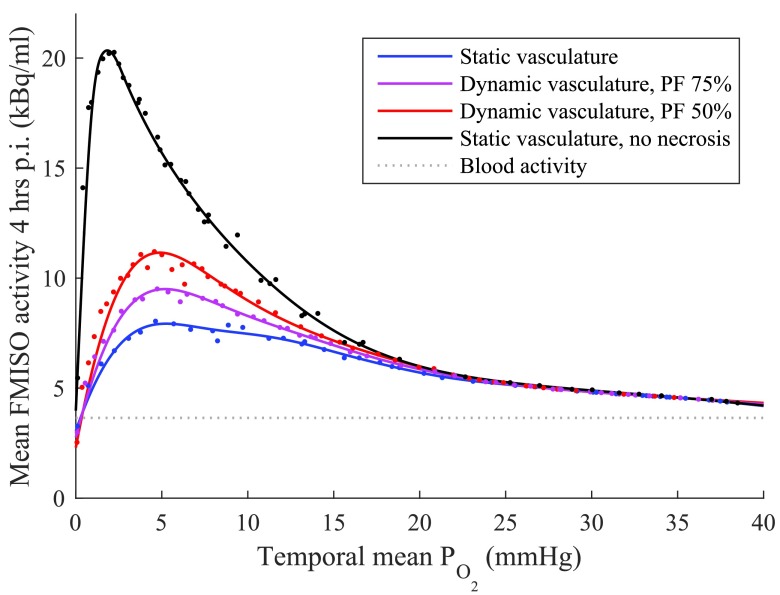
Volume-averaged temporal mean oxygen tension and FMISO activity 4 h p.i. for simulations with varying vascular density at different perfused fractions (PF, the average percentage of vessels perfused at any one time). Results for static vasculature without necrosis are also shown. Individual simulation results plotted as points; lines are smoothed spline fits to guide the eye. FMISO activity is attenuation corrected to time of injection.

Figure 3 shows the relationship between mean tissue ${{P}_{{{\text{O}}_{2}}}}$ and FMISO activity 4 h p.i. in 48 simulations with tissue of varying vascular density, under four different modelling assumptions. In all cases, a non-linear and non-monotonic (peaked) relationship is observed between the spatiotemporal mean ${{P}_{{{\text{O}}_{2}}}}$ and predicted FMISO signal. For simulations with static vasculature and no necrosis (i.e. *N*  =  0, regardless of local ${{P}_{{{\text{O}}_{2}}}}$), the maximal FMISO tissue:blood ratio (TBR) is greater than 5 and occurs at a ${{P}_{{{\text{O}}_{2}}}}$ of 1.7 mmHg. For simulations in which necrosis is considered, peak uptake is observed at 5 mmHg and the maximal TBRs are 3.0 and 2.6 for dynamic vasculature with average perfused fraction 50% and 75% respectively, and 2.2 for static vasculature. All simulations converge on a pseudo-linear decreasing relationship between FMISO signal and oxygenation in the range ${{P}_{{{\text{O}}_{2}}}}&gt;25$ mmHg (TBR  <  1.45).

**Figure 4.**
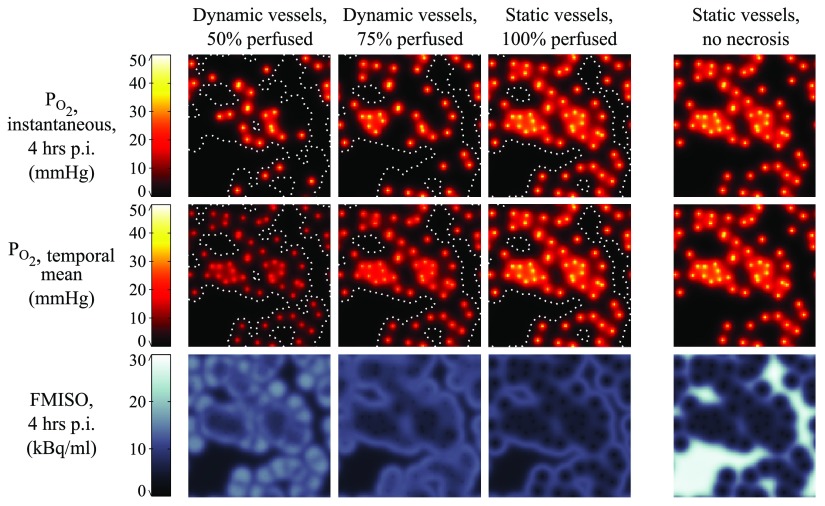
Simulated microscopic oxygen partial pressure (top: instantaneous; middle: temporal mean) and FMISO distribution maps (bottom). Simulations were performed in a vessel map with 60 vessels mm^−2^ and average instantaneous perfused fractions of 50%, 75% and 100%. White dotted lines denote isocontours of 50% necrosis. Results for static vasculature without necrosis are also shown. Maps represent an area measuring 1 mm  ×  1 mm.

Example 2D maps of instantaneous ${{P}_{{{\text{O}}_{2}}}}$, temporal mean ${{P}_{{{\text{O}}_{2}}}}$ and FMISO binding at 4 h are shown in figure 4. This figure illustrates the four modelling assumptions from figure 3 in a single vessel map with 60 vessels mm^−2^. The effect of increasing the average perfused fraction from 50% to 100% can be seen in the instantaneous ${{P}_{{{\text{O}}_{2}}}}$ maps (the number of oxygen sources increases) and in the temporal mean ${{P}_{{{\text{O}}_{2}}}}$ maps (a global increase in values). The vascular density in this scenario is relatively low, such that a halo of FMISO binding is seen on the fringe of vascularised sub-regions and around isolated vessels, beyond which little tracer is retained due to necrosis. For a given vascular map, the model predicts smaller regions of necrosis as perfused vessel fraction increases. If no necrosis is included in the model, strong binding is instead seen in the regions furthest from vessels. Animations depicting the evolution in FMISO and oxygen distributions over time are available in the supplementary material to this paper (stacks.iop.org/PMB/61/8596/mmedia).

Numerical data for the simulated vascular fraction, hypoxic fraction, necrotic fraction and FMISO TBR 1–4 h p.i. are given in table 4, at a range of tissue oxygenations and perfused fractions. In figure 3, the maximal TBR is seen to increase as the perfused vessel fraction decreases. This may be explained by reference to the necrotic fraction in the table, which represents the percentage of the volume in which FMISO is not able to be bound, and which decreases with perfused fraction at a given mean ${{P}_{{{\text{O}}_{2}}}}$.

**Table 4.** Summary statistics for simulations in tissue with a given spatiotemporal mean oxygen partial pressure $\langle {{P}_{{{\text{O}}_{2}}}}\rangle $ at varying perfused vessel fraction. Each table row corresponds to an individual simulation, where the vascular density was selected to achieve the specified spatiotemporal mean ${{P}_{{{\text{O}}_{2}}}}$. For example, the middle row of the top table should be interpreted as follows: with 50% of vessels perfused, a vascular fraction of 4.7% led to a spatiotemporal mean ${{P}_{{{\text{O}}_{2}}}}$ of 10 mmHg, hypoxic fraction of 40.4%  ±  5.9%, necrotic fraction 16.4% and FMISO TBR at 1 h/2 h/4 h of 1.36/1.73/2.55.

		VF	HF	NF	TBR-1h	TBR-2h	TBR-4h
	1 mmHg	0.6	93.6 ± 2.4	76.8	1.19	1.44	2.01
	5 mmHg	2.7	68.0 ± 4.2	32.4	1.45	1.95	3.03
$\langle {{P}_{{{\text{O}}_{2}}}}\rangle $	10 mmHg	4.7	40.4 ± 5.9	16.4	1.36	1.73	2.55
	20 mmHg	9.5	5.7 ± 2.4	6.4	1.15	1.30	1.63
	35 mmHg	31.4	0 ± 0	3.4	1.06	1.12	1.25
(a) 50% vessels perfused							

		VF	HF	NF	TBR-1h	TBR-2h	TBR-4h
	1 mmHg	0.4	93.4 ± 2.0	79.4	1.16	1.35	1.79
	5 mmHg	1.8	67.7 ± 2.7	42.6	1.31	1.64	2.40
$\langle {{P}_{{{\text{O}}_{2}}}}\rangle $	10 mmHg	3.4	38.1 ± 4.3	20.2	1.29	1.59	2.27
	20 mmHg	6.5	6.7 ± 1.5	7.6	1.15	1.29	1.62
	35 mmHg	21.5	0 ± 0	3.4	1.06	1.12	1.25
(b) 75% vessels perfused							

		VF	HF	NF	TBR-1h	TBR-2h	TBR-4h
	1 mmHg	0.2	96.0	87.6	1.06	1.18	1.40
	5 mmHg	1.3	71.1	46.9	1.27	1.56	2.20
$\langle {{P}_{{{\text{O}}_{2}}}}\rangle $	10 mmHg	2.5	39.9	22.1	1.26	1.53	2.12
	20 mmHg	4.7	2.4	7.1	1.13	1.26	1.55
	35 mmHg	15.7	0	3.3	1.06	1.12	1.25
(c) 100% vessels perfused							

		VF	HF	NF	TBR-1h	TBR-2h	TBR-4h
	1 mmHg	0.3	94.6	0	1.53	2.58	4.93
	5 mmHg	1.3	68.3	0	1.74	2.54	4.34
$\langle {{P}_{{{\text{O}}_{2}}}}\rangle $	10 mmHg	2.3	46.0	0	1.52	2.06	3.28
	20 mmHg	4.3	5.9	0	1.17	1.34	1.73
	35 mmHg	13.7	0	0	1.07	1.13	1.28
(d) 100% vessels perfused (no necrosis)							

*Key*.

VF  =  vascular fraction (percentage of tissue domain occupied by vessels, both perfused and unperfused).

HF  =  ‘hypoxic fraction’ (percentage of domain with instantaneous ${{P}_{{{\text{O}}_{2}}}}&lt;5$ mmHg), ±  corresponds to r.m.s. variation with time.

NF  =  necrotic fraction (percentage of cells assumed killed by hypoxic conditions).

TBR-1 h/2 h/4 h  =  domain-averaged FMISO tissue:blood ratio at 1/2/4 h p.i.

**Figure 5.**
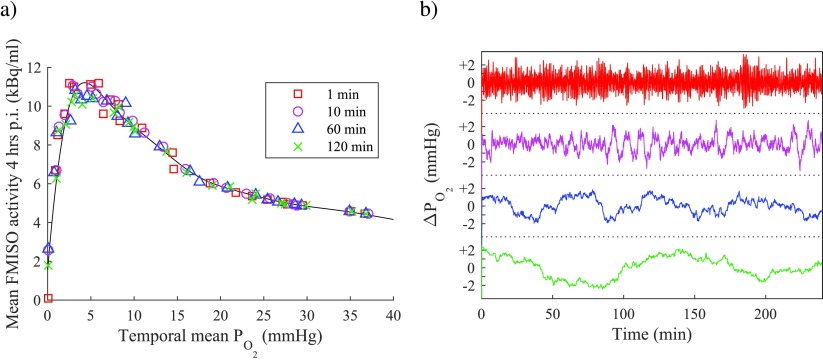
(a) Volume-averaged temporal mean oxygen tension and FMISO activity 4 h p.i for simulations in tissue with dynamic vasculature. Each symbol type represents simulations with a different average period of perfusion fluctuations (in the range 1 min–120 min), and average instantaneous perfused vessel fraction of 50%. Black line is a smoothing spline to guide the eye, fit to all data points. (b) Example time structures for variation in tissue mean ${{P}_{{{\text{O}}_{2}}}}$, around a value of 10 mmHg. Average period of fluctuations denoted by the same colour and position as in the legend of (a) i.e. top trace represents a period of 1 min, bottom trace represents 120 min.

The effect of varying the average period of perfusion fluctuations in the range 1–120 min is illustrated in figure 5. Figure 5(a) shows the relationship between spatiotemporal mean ${{P}_{{{\text{O}}_{2}}}}$ and FMISO TBR 4 hr p.i. for each time structure. No discernible differences in the relationship are observed as a result of changes in the time period. This can be explained in terms of the constituent parts of the FMISO binding rate expression in equation (4). The viable cell fraction *C* at a given temporal mean ${{P}_{{{\text{O}}_{2}}}}$ is independent of the fluctuation rate, since we assume that the necrotic fraction is a function of $\langle P\rangle $ alone (see equation (2)). The local free FMISO concentration *M*_*f*_ is relatively uniform across the domain for the majority of the imaging period, since the characteristic time for FMISO diffusion over typical intravascular distances ($\frac{{{x}^{2}}}{4D}$) is considerably shorter than the imaging period, and variations in the FMISO blood activity curve are gradual after the initial bolus. Finally, the radial ${{P}_{{{\text{O}}_{2}}}}$ profile (and therefore the binding parameter *k*_*b*_) is broadly the same for all vessels of a given perfusion state. The net result is that the FMISO bound 4 hr p.i. is essentially determined by the average number of vessels open and closed at any one time, rather than the time any particular vessel persists in each state.

#### 3.2.2. Dynamic uptake curves.

**Figure 6.**
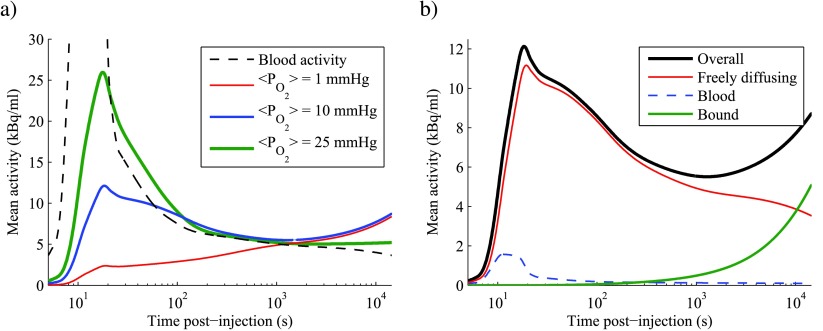
(a) Simulated time-activity curves for tissue with perfused fraction of 50% and three different vascular maps, leading to a range of temporal mean ${{P}_{{{O}_{2}}}}$ values. Plotted activity is a spatial average over the whole simulation domain, and is attenuation corrected to time of injection. Logarithmic time axis spans 4 h. Peak value of blood activity curve is 63.4 kBq ml^−1^ and occurs at 11 s. (b) Relative contributions of activity in each compartment of the model to the overall time-activity curve labelled $\langle {{P}_{{{O}_{2}}}}\rangle =10$ mmHg in (a). Animations showing the spatial distribution of FMISO in each compartment over time are available in the supplementary material.

Time-activity curves (TACs) are also simulated, allowing the investigation of model features that are relevant to dynamic PET studies. Figure 6(a) shows a plot of simulated TACs in tissue with average ${{P}_{{{\text{O}}_{2}}}}$ of 1, 10 and 25 mmHg, alongside the blood-activity curve used as an input. In the most hypoxic scenario, the observed TAC is essentially monotonically increasing. In the intermediate scenario, a peak in tissue activity occurs at the time of the FMISO bolus injection, but it is considerably broader than the equivalent feature in the blood activity curve and an accumulative behaviour is seen at later times. A more distinct peak is seen at the relatively well-oxygenated ${{P}_{{{\text{O}}_{2}}}}$ of 25 mmHg, followed by a consistent decay in tissue activity over the length of a typical PET study. Whilst the accumulation rate of bound FMISO appears to be increasing with time in figure 6, this is an artefact of the logarithmic time axis—the absolute binding rate is actually decreasing gradually as a result of the decaying concentration of tracer in blood.

Simulated 1 mmHg and 10 mmHg TACs follow almost identical trajectories at times greater than 10^3^ s, and it would therefore be impossible to discriminate between these two tissues by analysing static PET images acquired at late time-points. This observation can be explained intuitively with reference to table 4, noting that FMISO binds to regions that are hypoxic but not necrotic. In a very simplified model, tissue activity at late time-points will therefore be proportional to $\text{HF}-\text{NF}$, which is calculated to be approximately 20% at both 1 mmHg and 10 mmHg using the tabulated data for 50% vessels perfused.

It is also possible to extract the relative contributions of activity in the various compartments of the model as a function of time, providing insight into the shape of TACs. Figure 6(b) demonstrates this for a tissue TAC at 10 mmHg. At this ${{P}_{{{\text{O}}_{2}}}}$ only a small amount of activity directly originates from FMISO in blood (<20%, even at the peak of the activity curve) with the majority of signal arising in the gradually decaying diffusive compartment until approximately 10^4^ s. However, after approximately 10^3^ s, the greatest rate of change of tracer concentration is seen in the bound compartment, which therefore defines the shape of the TAC at later times. Animations depicting the spatial distribution of FMISO in each compartment are available in the supplementary material to this paper.

## 4. Discussion

### 4.1. Calibration of model parameters

Predictions of the calibrated oxygen and misonidazole binding model agree well with spheroid data published in the literature. Previously-reported ensemble oxygen consumption rates for EMT6 spheroids of diameter 300–1000 *μ*m are 1.80–6.44 ml${{}_{{{\text{O}}_{2}}}}$ · cm^−3^ · s^−1^ (Mueller-Klieser 1984) (approx. 9–21 mmHg s^−1^), and 4.1–7.9  ×  10^−17^ mol · cell · s^−1^ (Freyer and Sutherland 1985) (approx. 10–19 mmHg s^−1^)^3^3Assuming perfectly tessellating cells of volume 8  ×  8  ×  51 *μ*m^3^ (Chen *et al* 2011)., with higher consumption rates being observed in smaller spheroids. For comparison, spheroids simulated using equation (1) and the calibrated values of *q*_max_ and *P*_50,*q*_ from table 3 have volume-averaged consumption rates of 12–17 mmHg · s^−1^ over the same diameter range. Hypoxic necrosis data for EMT6 cells is available in the form of the thickness of viable rim in large spheroids, which is strongly influenced by glucose concentration in the medium but is relatively insensitive to spheroid diameter (Freyer and Sutherland 1986, Mueller-Klieser *et al* 1986). At atmospheric oxygen levels and physiological glucose concentrations, experimental data suggest a viable rim thickness for EMT6 cells of 190–220 *μ*m (Freyer and Sutherland 1985, Luk and Sutherland 1987): in spheroids simulated with the calibrated model this would be the case over the realistic diameter range 700–1200 *μ*m. EMT6 is a murine breast cancer cell line, but the oxygen consumption rates found here also agree well with direct measurements of oxygen consumption in the human breast cancer cell line MDA-MB-468 (18.08  ±  4.53 mmHg s^−1^) (Grimes *et al* 2016a).

FMISO binding parameters are model-specific, but agreement between similar parameters nevertheless provides confidence in the calibrated model. The calibrated model value for 50% maximal binding in EMT6 cells was ${{P}_{50,b}}=1.4\text{mmHg}$. Casciari identified an oxygen concentration for half-maximal FMISO binding of 2710 ppm (${{P}_{{{\text{O}}_{2}}}}$ approx. 2.1 mmHg) in V79 cells (Casciari and Rasey 1995); Rasey found values in the range 1000–2000 ppm (${{P}_{{{\text{O}}_{2}}}}$ approx 0.8-1.5 mmHg) in four different cell lines, and suggests that this parameter may be cell line-dependent (Rasey *et al* 1989). The maximal FMISO binding rate without necrosis has previously been reported as $8.7\times {{10}^{-4}}{{\text{s}}^{-1}}$, found by fitting Casciari’s model to temporal uptake measurements in V79 monolayers (Casciari and Rasey 1995). The equivalent parameter in this model is ${{k}_{b,0}}=4.5\times {{10}^{-4}}{{\text{s}}^{-1}}$, which is smaller but of the same order of magnitude. There is no simple explanation for this discrepancy, but it might partly be attributable to cell-line dependence, and to differences in cell packing density between monolayer and spheroid cultures. Including the term for necrosis, the maximal predicted binding rate in tissue is $1.2\times {{10}^{-4}}{{\text{s}}^{-1}}$, which is comparable to that suggested independently by Mönnich *et al* (2011) (calculated as $1.3\times {{10}^{-4}}{{\text{s}}^{-1}}$).

### 4.2. Comparison of simulated tumour FMISO uptake to ***in vivo*** data

The clinical relevance of absolute FMISO binding rates calculated by the parameterised model can be assessed by comparing simulated tissue-to-blood ratios (TBRs) to *in vivo* measurements. This model was derived using data from EMT6 cells, a breast cancer line. In one of the few studies reporting on FMISO uptake in breast tumours, maximal TBRs at 2 h p.i. were reported for 7 patients, with an average of 1.5 (range 0.9–2.6) (Rajendran 2004). Similar TBR ranges were reported in the same paper for head and neck tumours and sarcomas. Our simulated TBRs at 2 h p.i. range from 1.3 (in the well-oxygenated case) up to 1.6–2.0 (at the peak, dependent on perfused fraction) for the model with necrosis, and up to 2.8 for the model without necrosis. In general, most of the reported range can be accounted for by the model, if we accept the possibility of variations in the propensity for necrosis in the clinical tumours. The exception is the small numbers of TBRs below 1, which cannot be accounted for in the model as formulated, but could be explained by the presence of significant amounts of non-diffusing material within the image voxel. Rajendran reported a notably higher range for maximal TBRs at 2 h p.i. in brain (mean 2.4, range 1.7–2.9). It is suggested that a different model may be necessary to quantitatively match brain data, since the cerebral environment is markedly different to other areas of the body.

Simulation results show a turnover in FMISO uptake as a function of tissue oxygenation, as illustrated in figure 3: no uptake is seen in complete anoxia, sharply rising to a peak at approximately 5 mmHg, followed by a gradual decrease at higher ${{P}_{{{\text{O}}_{2}}}}$. Comparable pre-clinical and clinical data is available from studies in which tumour hypoxic fractions have been measured before or after FMISO PET imaging.

Precisely-guided animal experiments, employing microPET and fluorescence-based oxygen measurements, showed a broadly monotonic decreasing relationship between the two variables in all but the most hypoxic regions (Chang *et al* 2009). However, considerable variation in FMISO uptake was seen in regions with ${{P}_{{{\text{O}}_{2}}}}$ measurements close to 0 mmHg—this could be consistent with varying levels of hypoxic necrosis at low ${{P}_{{{\text{O}}_{2}}}}$, but the data is too scattered to be viewed as confirmation or contradiction of the turnover relationship observed in the simulations. A proof-of-concept study using a different robotic platform illustrated a turnover relationship along one track through a single tumour (Hsu *et al* 2011).

Clinical studies in head and neck cancer patients have suggested a monotonic increasing relationship between FMISO and hypoxia at hypoxic fractions  <70% (Zimny *et al* 2006, Gagel *et al* 2007). The results of our simulations are consistent with this observation, since the data in table 4 indicates that the turnover in the oxygenation-retention curve occurs at HF close to 70%. Furthermore, these authors found greatest departures from a monotonic FMISO versus hypoxia relationship in small, highly-necrotic tumours. In another head and neck cohort, Mortensen and colleagues compared measured hypoxic fractions with those estimated from FMISO images, assuming a linear mapping between TMR (in the range 1.0–3.7) and hypoxic fraction (corresponding range 0–100%) (Mortensen *et al* 2010). Whilst no statistically significant correlation was reported, it is notable that the greatest FMISO signals were observed in regions of intermediate (as opposed to complete) measured hypoxia, and some of the illustrated data is consistent with a turnover at very low mean ${{P}_{{{\text{O}}_{2}}}}$.

### 4.3. Clinical implications of model predictions

It has already been established that a broad range of cellular ${{P}_{{{\text{O}}_{2}}}}$ values exist in tissue regions of PET voxel size (Busk *et al* 2008, 2009), and that FMISO binding is likely to be similarly heterogeneous (Kelly and Brady 2006, Mönnich *et al* 2011, Mönnich *et al* 2012, Mönnich *et al* 2013). Our results support those observations, and emphasize that there is a possibility of millimetre-sized tumour regions containing well-oxygenated, hypoxic and necrotic regions over a wide ${{P}_{{{\text{O}}_{2}}}}$ range. As a result, FMISO uptake at late time points may be dependent upon the competing effects of hypoxia (increasing binding) and necrosis (decreasing binding)—both of these factors should therefore be considered when developing a strategy for hypoxia assessment using FMISO PET. Simulated time activity curves suggest that perfusion information in the early phase of a dynamic PET study ($\lesssim $${{10}^{3}}\text{s}$) may enable the differentiation of well-oxygenated and highly-necrotic regions. Full pharmacokinetic modelling may not be necessary: for example, F_H/P_, the ratio of activity in a voxel at 4 h to its average activity in the first 15 min of the study, is a promising metric proposed by Mönnich *et al* (2013).

At present, tumour hypoxic volumes are often assessed by defining thresholded regions on a static PET image, but there is not consensus on the precise threshold applicable (typically in the range 1.2–1.5) or the ideal delay between contrast injection and image acquisition (Peeters *et al* 2015). Computational modelling can provide estimates of the oxygenation levels to which these thresholds correspond. Specific parameterisation of the model for a particular tumour type will provide the most robust estimates. With the generic model described here, TBR  >  1.4 at 4 h p.i. corresponds to tissue with temporal mean ${{P}_{{{\text{O}}_{2}}}}$ above 0.1–1.3 mmHg and below 23 mmHg, whilst tumour:normal tissue ratio  >  1.3 is predicted in regions above 0.1–1.1 mmHg and below 27 mmHg^4^4Under the assumption that normal tissue (e.g. muscle) is modelled by the maximally-oxygenated simulation.. The upper limits are quite robust to differences in vessel perfusion status, whilst the lower limits are dependent on the level of hypoxic necrosis.

## 5. Conclusions

We have presented a computational model of FMISO binding in tumour tissue, which has been calibrated using experimental data, and which predicts tumour:blood ratios that match clinical data over a realistic range of ${{P}_{{{\text{O}}_{2}}}}$. Simulations show a non-linear, non-monotonic relationship between millimetre-scale tissue oxygenation and FMISO uptake at 4 h, with a sharp fall-off at low oxygen levels due to necrosis.

Our model has, for the first time, simulated the effects of temporally-incoherent cyclic variations in perfusion on FMISO contrast. Results suggest that the signal observed at late time-points does not specifically relate to ‘chronic’ or ‘acute’ hypoxia, but is instead representative of the time-averaged oxygenation during the imaging study. Transient perfusion may affect the maximum possible signal by increasing the number of viable cells available to bind tracer at a given spatiotemporal mean ${{P}_{{{\text{O}}_{2}}}}$ : more tissue will be exposed to oxygen over a long time period, and necrosis may therefore be more limited. Varying the average timescale of perfusion fluctuations in the range 1–120 min does not seem to appreciably affect the observed binding at late time-points. It should, however, be noted that correlated variations in perfusion may affect the time-averaged oxygenation and therefore cause changes in the FMISO signal, such as those observed by Mönnich *et al* (2012).

Since the model predicts that FMISO contrast arises in cells that are hypoxic but not yet necrotic, static FMISO PET images may be confounded by the effects of necrosis. A dose-painting strategy that is driven by a one-to-one mapping between static FMISO imaging and dose^5^5E.g. defining biological treatment volume by thresholding; linear escalation of dose according to voxel uptake. will therefore risk underdosing small populations of hypoxic cells in mostly necrotic regions. However, our results indicate that additional information present in dynamic PET scans may allow the differentiation of necrotic and oxic signals, in concordance with previous authors (Mönnich *et al* 2013). We suggest that a robust dose-painting strategy is likely to be based on dynamic PET analysis, such as the methodology described by Thorwarth (2007a, 2007b, 2008).

This work suggests that quantifying necrosis may be an important step in interpreting image contrast in FMISO PET, and potentially other hypoxia imaging modalities. Little data is available regarding the quantitative relationship between tissue oxygenation and necrosis, and how that might be affected by temporal variations in oxygenation. Biological factors beyond the scope of this model (e.g. glucose availability) may also modulate necrosis and therefore the FMISO signal. Further experimental work along these lines would be a useful contribution to our understanding of hypoxia imaging.

## Figures and Tables

**Figure A1.**
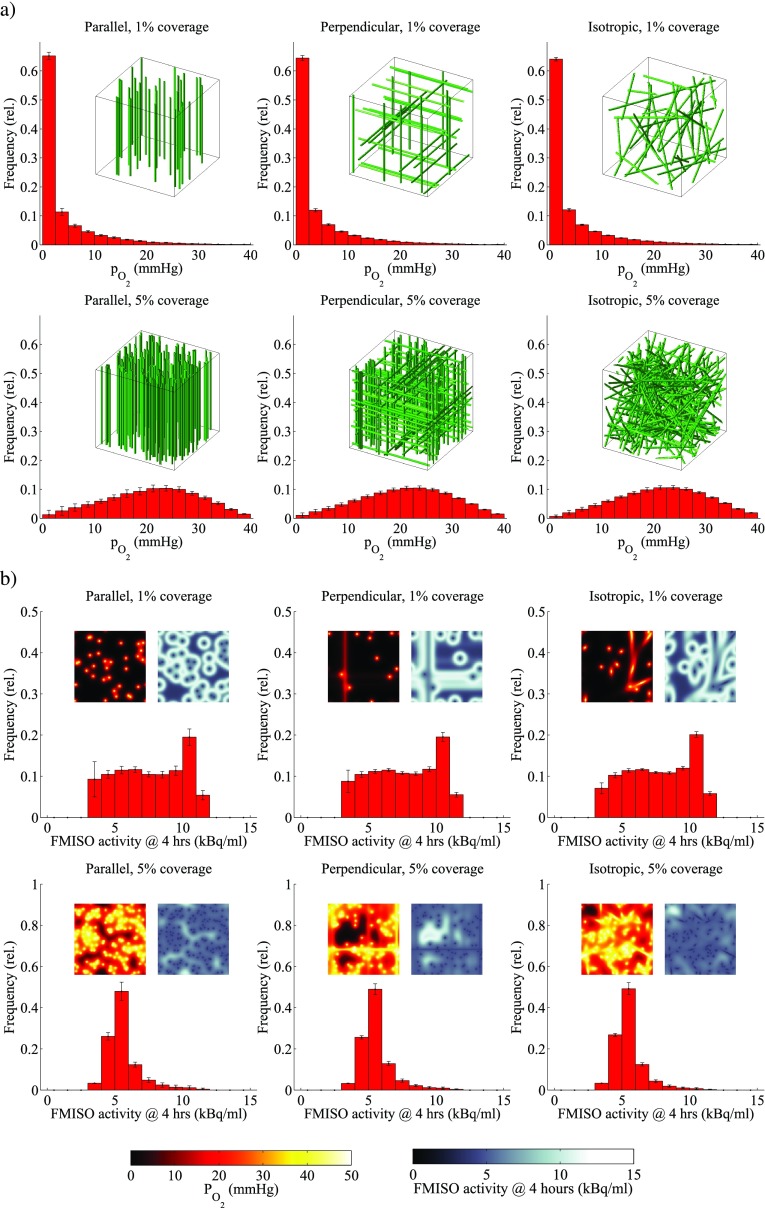
(a) Histograms representing the simulated oxygen partial pressure (${{P}_{{{\text{O}}_{2}}}}$) distributions in tissue with three-dimensional vessel maps measuring 1 mm^3^ and vascular volume fractions of 1% and 5%. Three vessel orientations are examined (parallel, perpendicular and isotropic), and example volume renderings of the vessel maps are inset. Bin heights represent the mean of 20 simulations, and error bars denote  ±1 standard deviation. (b) Histograms representing the simulated FMISO activity distributions in the same 3D volumes as (a), 4 h after injection. Insets show example 2D slices of ${{P}_{{{\text{O}}_{2}}}}$ (left) and FMISO (right) distributions. Bin heights represent the mean of 20 simulations, and error bars denote  ±1 standard deviation.

**Figure B1.**
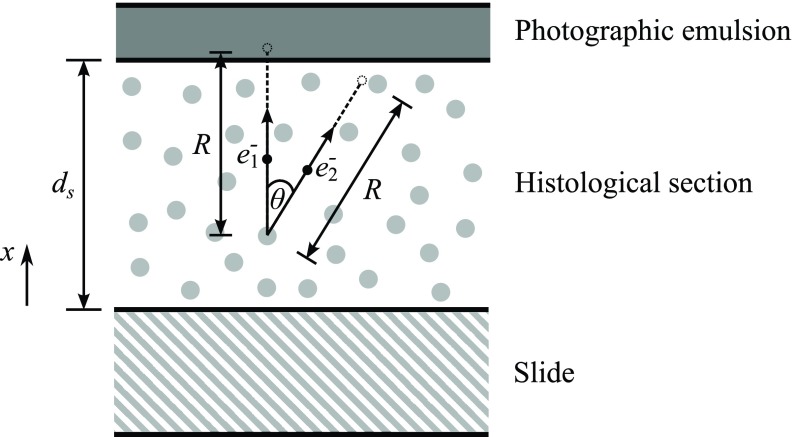
Geometry for the autoradiography quantification model. Two possible initial trajectories (${{e}^{-}}_{1}$ and ${{e}^{-}}_{2}$) are shown for electrons of equal energy and thus equal range *R*. ${{e}^{-}}_{1}$ will be detected as a grain activation, but ${{e}^{-}}_{2}$ will never reach the emulsion since $R\cos \theta &lt;{{d}_{s}}$.

## Appendix A. Oxygen and FMISO diffusion in 2D versus 3D

Espinoza *et al* (2013) have previously compared simulated ${{P}_{{{\text{O}}_{2}}}}$ histograms in vascular maps where cylindrical blood vessels run parallel to each other (equivalent to a 2D simulation geometry) or are randomly oriented along one of three mutually perpendicular directions. They observed no statistically significant differences when comparing the average hypoxic volume (defined as ${{P}_{{{\text{O}}_{2}}}}&lt;5$ mmHg) in twenty simulations of each model with the same vascular volume, and conclude that 3D simulation is unnecessary. That result is confirmed below, considering the additional possibility of an isotropic angular distribution such that vessels may travel at oblique angles to each other. We also perform a similar comparison for the FMISO diffusion and binding model.

### A.1. Methods

New oxygen and misonidazole diffusion simulations were carried out in a 1 mm^3^ cubic domain consisting of cubic voxels with side length 10 *μ*m and periodic boundary conditions. Vessels were modelled by rasterized representations of a cylinder with radius 10 *μ*m calculated to 0.1% precision. The directions of cylinder axes were sampled from the relevant p.d.f. (probability density function) given in table A1, and origin points were sampled from a uniform spatial p.d.f. The parallel vessel arrangement can be considered equivalent to a 2D model.

In order to ensure that all simulations had equal vessel coverage, vessels were added to the vascular map *V* one-at-a-time until the calculated relative vascular volume $\left(\frac{1}{{{N}_{i}}}{\sum}_{i}{{V}_{i}}\right)$ exceeded a target fraction. The finite-difference simulation method and model parameters were identical to those used for static vasculature in the main text, except for the use of a three-dimensional discrete Laplace operator.

### A.2. Results

Figure A1 shows average steady-state ${{P}_{{{\text{O}}_{2}}}}$ and 4 h FMISO histograms for 20 simulations with each vessel arrangement and coverage fractions of 1% and 5%. Only very minor variations between distributions are apparent, the statistical significance of which were assessed using the chi-square test on a bin-by-bin basis. Significant differences (at the 95% level) in the oxygen histograms were only seen for bins above 30 mmHg. It is suggested that these small differences arise due to a slight bias in the vessel seeding method: an additional parallel or perpendicular vessel necessarily spans the entire simulation volume, whereas isotropic vessels may only span a fraction of the volume. No significant differences were seen in the FMISO histograms. We conclude that 2D simulations are sufficiently accurate to justify their use in this study.

It should be noted that the whole vascular map has been pre-determined and used as an input to these simulations, a situation which is not typically possible for experimental data analysis. In addition, the comparisons have considered summary histograms rather than local spatial distributions of oxygen and tracer. Grimes *et al* (2016b) have reported considerable variability in oxygen distributions calculated by sampling single 2D slices from a 3D vessel network, as would be the case if a single histological section is used to estimate the vasculature. Caution should therefore be exercised in performing 2D analysis of experimental data.

**Table A1.** Probability density functions (p.d.f.) sampled in order to model each vessel arrangement. $\delta (x)$ is the Dirac delta function.

Vessel arrangement	Azimuthal p.d.f.	Polar p.d.f.
Parallel	$f(\theta )=\delta (\theta )$	$f(\phi )=\delta (\phi )$
Perpendicular	$f(\theta )=\frac{1}{4}{\sum}_{n=0}^{3}\delta \left(\theta -\frac{n\pi}{2}\right)$	$f(\phi )=\frac{1}{2}\left[\delta (\phi )+\delta \left(\phi -\frac{\pi}{2}\right)\right]$
Isotropic	$f(\theta )=\left\{\begin{array}{*{35}{l}} \frac{1}{2\pi} &amp; 0\leqslant \theta &lt;2\pi \\ 0 &amp; \text{otherwise} \end{array}\right. $	$f(\phi )=\left\{\begin{array}{*{35}{l}} \frac{1}{2}\sin \phi &amp; 0\leqslant \phi &lt;\pi \\ 0 &amp; \text{otherwise} \end{array}\right. $

## Appendix B. Quantification of autoradiography

A method for the quantification of autoradiography with tritium-labelled molecules has previously been reported by Kellerer *et al* (1993). We follow that work but with an explicit consideration of the isotropic emission of electrons, as expected in the beta decay of unpolarised nuclei. A similar method could be followed for alternative labels (e.g. ^14^C).

The quantity ${{\eta}_{a}}$, autoradiographic efficiency, for a section of thickness *d*_*s*_ will be defined as follows:

\begin{eqnarray*}{{\eta}_{a}}\left({{d}_{s}}\right)={\int}_{0}^{{{d}_{s}}}{\int}_{0}^{{{E}_{\text{max}}}}{\int}_{-\pi}^{\pi}P(E)\centerdot H\left(R(E)\centerdot \cos \theta -x\right)\text{d}\theta \text{d}E\text{d}x\end{eqnarray*}

where *P*(*E*) is the beta decay energy spectrum (normalised to integral 1), *R*(*E*) is the range of an electron with energy *E*, *θ* is the angle between the emitted electron’s initial trajectory and the section’s surface normal, and *H*(*x*) is the Heaviside step function. The geometry is illustrated in figure B1.

${{\eta}_{a}}$ can be interpreted as the proportion of electrons detectable at the surface of the sample relative to all electrons emitted within the sample. We also define a radiolabelling efficiency ${{\eta}_{l}}$, which is the ratio of the specific activity of the labelled tracer ${{a}_{\rho}}$ (in Bq g^−1^) to the theoretical maximum (when each molecule of tracer is bonded to a single label):

\begin{eqnarray*}{{\eta}_{l}}={{a}_{\rho}}\centerdot \left(\frac{{{t}_{\frac{1}{2}}}}{{{N}_{A}}\centerdot \log 2}\right)\centerdot \frac{m}{{{N}_{l}}}\end{eqnarray*}

where ${{t}_{\frac{1}{2}}}$ is the label’s half-life in seconds, *N*_*A*_ is the Avogadro constant, *m* is the mass of the tracer in a.m.u, and *N*_*l*_ is the number of labels per tracer molecule.

Under the assumption that a single electron track reaching the emulsion activates exactly one grain, a measured areal density of activated grains *σ* can be related to $\bar{c}$, the molar concentration of tracer in the sample averaged along the *x* direction.

\begin{eqnarray*}\sigma ={{\eta}_{l}}{{\eta}_{a}}\centerdot \bar{c}{{N}_{A}}{{d}_{s}}\centerdot \frac{\log 2\centerdot {{t}_{\text{exp}}}}{{{t}_{\frac{1}{2}}}}\end{eqnarray*}

\begin{eqnarray*}\bar{c}=\frac{1}{{{\eta}_{l}}{{\eta}_{a}}}\centerdot \frac{\sigma}{{{N}_{A}}{{d}_{s}}}\centerdot \frac{{{t}_{\frac{1}{2}}}}{\log 2\centerdot {{t}_{\text{exp}}}}\end{eqnarray*}

where *t*_exp_ is the exposure time in seconds. To obtain a concentration in $\text{mol}\centerdot \text{d}{{\text{m}}^{-3}}$, *σ* is specified in $\text{d}{{\text{m}}^{-2}}$ and *d*_*s*_ in dm. In the special case of a sample that is thicker than ${{R}_{\text{max}}}\equiv R\left({{E}_{\text{max}}}\right)$, the maximum range of an emitted electron:

\begin{eqnarray*}\bar{c}=\frac{1}{{{\eta}_{l}}{{\eta}_{a}}\left({{R}_{\text{max}}}\right)}\centerdot \frac{\sigma}{{{N}_{A}}{{R}_{\text{max}}}}\centerdot \frac{{{t}_{\frac{1}{2}}}}{\log 2\centerdot {{t}_{\text{exp}}}}.\end{eqnarray*}
